# Which symptom to address in psychological treatment for cancer survivors when fear of cancer recurrence, depressive symptoms, and cancer-related fatigue co-occur? Exploring the level of agreement between three systematic approaches to select the focus of treatment

**DOI:** 10.1007/s11764-023-01423-z

**Published:** 2023-08-01

**Authors:** Susan J. Harnas, Sanne H. Booij, Irene Csorba, Pythia T. Nieuwkerk, Hans Knoop, Annemarie M. J. Braamse

**Affiliations:** 1 Medical Psychology, Amsterdam UMC, University of Amsterdam, Meibergdreef 9, Amsterdam, The Netherlands; 2 Cancer Treatment and Quality of Life, Cancer Center Amsterdam, Amsterdam, Netherlands; 3 Mental Health, Amsterdam Public Health, Amsterdam, The Netherlands; 4 Department of Psychiatry, Interdisciplinary Center Psychopathology and Emotion Regulation, University Medical Center Groningen, University of Groningen, Groningen, The Netherlands; 5 Center for Integrative Psychiatry, Lentis, Groningen, The Netherlands; 6 Infectious Diseases, Amsterdam Institute for Infection and Immunity, Amsterdam, Netherlands

**Keywords:** Cancer survivors, Fear of cancer recurrence, Depressive symptoms, Cancer-related fatigue, Treatment focus, Ecological momentary assessments (EMA)

## Abstract

**Purpose:**

To investigate the extent to which three systematic approaches for prioritizing symptoms lead to similar treatment advices in cancer survivors with co-occurring fear of cancer recurrence, depressive symptoms, and/or cancer-related fatigue.

**Methods:**

Psychological treatment advices were was based on three approaches: patient preference, symptom severity, and temporal precedence of symptoms based on ecological momentary assessments. The level of agreement was calculated according to the Kappa statistic.

**Results:**

Overall, we found limited agreement between the three approaches. Pairwise comparison showed moderate agreement between patient preference and symptom severity. Most patients preferred treatment for fatigue. Treatment for fear of cancer recurrence was mostly indicated when based on symptom severity. Agreement between temporal precedence and the other approaches was slight. A clear treatment advice based on temporal precedence was possible in 57% of cases. In cases where it was possible, all symptoms were about equally likely to be indicated.

**Conclusions:**

The three approaches lead to different treatment advices. Future research should determine how the approaches are related to treatment outcome. We propose to discuss the results of each approach in a shared decision-making process to make a well-informed and personalized decision with regard to which symptom to target in psychological treatment.

**Implications for Cancer Survivors:**

This study contributes to the development of systematic approaches for selecting the focus of psychological treatment in cancer survivors with co-occurring symptoms by providing and comparing three different systematic approaches for prioritizing symptoms.

## Introduction

Fear of cancer recurrence, depressive symptoms, and cancer-related fatigue are common symptoms among cancer survivors. A recent meta-analysis found that more than half (59%) of cancer survivors reported moderate to severe levels of fear of cancer recurrence [1]. It is further estimated that approximately 1 in 4 cancer survivors experience moderate to severe depressive symptoms and approximately 1 in 4 report severe cancer-related fatigue [2–4]. Symptoms such as fatigue, depression, and anxiety often co-occur, and it is known that co-occurrence of symptoms can worsen patient outcomes [5–9].

For treating individual symptoms, use of evidence-based symptom-specific interventions is indicated, such as cognitive behavioral therapy for depressive symptoms and fatigue and meta-cognitive therapy for fear of cancer recurrence [9–13]. However, in case symptoms co-occur, it is not straightforward how to prioritize symptoms and therefore how to determine which symptom-specific intervention to apply first. A psychological treatment based on a transdiagnostic approach could be a possible solution for this problem. A transdiagnostic psychological treatment targets underlying mechanisms common to several psychological symptoms, which provides an opportunity to address multiple symptoms at the same time [14]. However, thus far, the evidence for transdiagnostic interventions for co-occurring symptoms in cancer survivors is limited [15, 16], while there are evidence-based symptom-specific interventions focusing on depression, fatigue, and fear of cancer recurrence in cancer survivors [9–12].

In clinical practice, therapists use their clinical judgment to advice which intervention is most appropriate, probably in interaction with patients in a shared decision process. Although this is evidently a valuable approach, it contains biases, e.g., caused by the therapist’s knowledge of and training in specific treatment protocols and is often not systematic nor evidence-based [17–20]. The decisions mainly rely on intuition and heuristics instead of algorithmic processing. Indeed, clinicians do not have a large body of evidence to base their decision on. In many intervention studies, participants are selected based on stringent criteria, excluding or neglecting co-occurring symptoms as the aim is to achieve a homogenous group of patients [21–23]. A criticism against these research designs is a lack of external validity as most patients report more than one symptom [19, 24].

There is a need for research that builds a knowledge base on how to prioritize symptoms in case of co-occurrence, as co-occurrence is often the case in clinical practice. In the end, the ultimate aim in research as well as in clinical practice is to choose the psychological treatment which leads to optimal treatment outcomes, lowest drop-out rates, and highest patient satisfaction. To achieve this for patients with co-occurring symptoms, firstly different systematic and reliable approaches to prioritize symptoms need to be identified, and the extent to which these approaches lead to similar treatment advices should be established. If the approaches lead to different treatment advices, a next step would be to investigate which approach predicts the best outcomes.

In this study, we applied three different systematic approaches for prioritizing symptoms and determined the level of agreement between these approaches with regard to which symptom-specific intervention to apply. We studied this in cancer survivors with co-occurring fear of cancer recurrence, depressive symptoms, and cancer-related fatigue.

A first approach for prioritizing symptoms is to assess the preference of the patient. In a previous meta-analysis, in which the impact of accommodating patient preference in psychotherapy was investigated, multiple ways of measuring patient preference were discussed [25]. The most popular measure was to directly ask the patients what treatment they prefer to receive. The meta-analysis showed that patients receiving their preferred therapy conditions were less likely to drop out of therapy prematurely and showed greater improvements in treatment outcomes than patients not receiving their preferred therapy conditions. In another meta-analysis, the positive association between patient preference and fewer drop-outs was confirmed [26]. There was however no evidence of a significant association with other treatment outcomes.

A second approach for prioritizing symptoms is to compare the severity of the symptoms and let the most severe symptom determine the treatment advice. A similar approach provided preliminary evidence that treatment based on this approach may be more efficient [27, 28]. In addition, this approach aligns well with so-called routine outcome measurements (ROM), which are implemented in various disciplines in clinical practice. With ROM, outcome measurements on symptom severity are regularly assessed with the aim to evaluate patients’ progress in treatment and, if necessary, adapt treatment [29].

A third and relatively recent approach is the assessment of temporal dynamics between symptoms. Research has shown that psychological symptoms fluctuate over time, can covary, and show temporal precedence, i.e. one symptom changes before the other does [30, 31]. Patients differ in the dynamics between symptoms, which can be visualized with individual symptom networks [8, 30, 31]. Within a symptom network of an individual patient, temporal precedence and bidirectional associations between symptoms can be investigated with individual time series analyses based on ecological momentary assessments (EMA) [31]. Treating the symptom which precedes other symptoms may make treatment more efficient as positive effects on co-occurring symptoms can be expected. Various studies have applied this approach or called for its application [30, 32, 33]. In a previous study, we also applied this approach in cancer survivors with cancer-related fatigue [34]. Results showed that, for example, fear of cancer recurrence can precede fatigue in one patient but not in another.

Altogether, in this study, we aimed to investigate the extent to which three systematic approaches for prioritizing symptoms lead to similar treatment advices on which symptom to treat in cancer survivors with co-occurring fear of cancer recurrence, depressive symptoms, and/or cancer-related fatigue. The three systematic approaches we investigated were based on [1] patient preference, [2] symptom severity, and [3] temporal precedence.

## Methods

This paper is reported in accordance with the STROBE guidelines for cross-sectional studies [35].

### Study design

The current study is an explorative cross-sectional study using baseline data from an ongoing randomized controlled trial (RCT), the MATCH study. In the MATCH study, the efficacy of a personalized symptom-specific intervention is compared to a standard symptom-specific intervention in cancer survivors with fear of cancer recurrence, depressive symptoms, or fatigue. The trial is open for inclusion since February 2019. A detailed description of the MATCH study is published elsewhere [36].

### Setting and procedure

Patients were referred to the MATCH study via [1] their treating physician at the outpatient cancer clinics of four academic hospitals and four general hospitals in the Netherlands, [2] self-referral, and [3] a psycho-oncological mental health care center. The mental health care center referred patients who were on the waiting list for an intake.

After referral, eligibility for the RCT was assessed. Medical information regarding the cancer diagnosis and cancer treatment was gathered, and patients completed screening questionnaires (see below). Subsequently, patients completed the baseline assessment, after which randomization determined allocation to either the personalized or the standard treatment arm.

### Participants

As part of the inclusion criteria for the MATCH study, all patients completed their primary, curative cancer treatment at least 6 months before screening with a maximum of 5 years, were ≥ 18 years old, were able to speak and read Dutch, had no disease activity at the time of inclusion, were not currently receiving psychological or psychiatric treatment, and reported clinically relevant levels of fear of cancer recurrence and/or depressive symptoms and/or fatigue, from which they experienced functional impairments [36]. For the present study, we only included patients who reported clinically relevant levels for two or more of the abovementioned symptoms. This meant that patients at baseline scored above the cut-off value on at least two of the three symptom questionnaires. In addition, we only included patients from the personalized treatment arm, as in the standard arm EMA was not used.

### Variables and measurement

Sociodemographic and medical information was collected per participant. In addition, we collected patient preference, symptom severity, and EMA to search for temporal precedence (see below for further specifications). Sociodemographic information, medical information, patient preference, and symptom severity were assessed through online self-report questionnaires, which were hosted by Castor EDC (https://data.castoredc.com). EMA were offered through an online electronic diary system (http://roqua.nl). In both cases, it was not possible to skip questions.

#### Sociodemographic information

The following sociodemographic information was collected based on self-report: gender, age, educational level, whether the patient has a partner, and whether the patient had previously received psychological or psychiatric care.

#### Medical information

The following information was extracted from patients’ medical files: cancer diagnosis, type of cancer treatment, time since cancer diagnosis, time since completion of cancer treatment, and comorbid conditions.

#### Patient preference

By a multiple choice question developed specifically for the MATCH study, patients were asked after their preferred focus for psychological treatment: “If you had to choose, what is the most limiting symptom for which you would like to receive help from a psychologist?”. Patients could select fear of cancer recurrence, depressive symptoms, fatigue, or none of the above.

#### Symptom severity — fear of cancer recurrence

Level of fear of cancer recurrence was measured with the 6-item Cancer Worry Scale (CWS). Each item can be answered on a 4-point Likert scale. Total scores range from 6 to 24. The CWS has good construct validity, convergent and divergent validity, and high internal consistency (in the current sample: *⍺* = 0.91) [37]. A cut-off value of ≥ 10 is optimal for identifying highly fearful patients [37].

#### Symptom severity — depressive symptoms

Levels of depressive symptoms were measured with the 7-item Beck Depression Inventory for Primary Care (BDI-PC), also known as the Beck Depression Inventory-Fast Screen (BDI-FS). Each item can be answered on a 4-point Likert scale and total scores can range from 0 to 21. The BDI-PC has been shown to be a valid and reliable instrument to screen for depressive disorders in patients with somatic diseases [38, 39]. The BID-PC has high internal consistency (in the current sample: *⍺* = 0.61) and displayed convergent validity. A cut-off value of ≥ 4 is optimal to detect clinically relevant depressive symptoms [38, 39].

#### Symptom severity — fatigue

Level of fatigue severity was measured with the subscale 'fatigue severity' of the Checklist Individual Strength (CIS) [40]. The subscale consists of 8 items, and each item can be answered on a 7-point Likert scale. Total scores can range from 8 to 56. Based on previous research, a cut-off value of ≥ 35 indicates presence of severe fatigue [40–42]. The CIS has been established as a valid and reliable measure with high internal consistency (in the current sample: *⍺* = 0.79) [40, 43, 44].

#### Temporal precedence — EMA

All patients completed EMA at the start of treatment. Assessments were offered five times a day during 14 consecutive days at fixed intervals (every 3 hours). The exact time points of the assessments were personalized to the sleep–wake cycle of each patient. Patients received a text message on their mobile phone, which included a link to a questionnaire. In this questionnaire, patients were asked to indicate (per symptom) the extent to which they experienced fear of cancer recurrence and feelings of depression and fatigue in the last 3 hours. The three questions, used for this study, were a selection of the original questionnaire [36]. Each item was answered on a continuous scale (0–100). Patients were asked to answer the questions as soon as they received the alert on their mobile phone. If the patient did not complete the questionnaire within 30 minutes, they received a reminder text message. If the patient did not complete the questionnaire within 60 minutes, the link to the questionnaire expired and could not be accessed anymore (labeled as missing measurement).

### Data preparation

To calculate the level of agreement between the three systematic approaches, we first established per approach which symptom-specific treatment was indicated, i.e. the treatment advice.

#### Treatment advice based on patient preference

Self-reported patient preference determined the treatment advice.

#### Treatment advice based on symptom severity

The symptom level which deviated the most from the mean of a reference group determined the treatment advice. To compare symptom severity scores, we transformed each depression, fear of cancer recurrence, and fatigue score into a standardized *z*-score per patient. The symptom with the highest *z*-score determined the treatment advice. The difference between the two highest z-scores had to be at least 0.2 or more, i.e. indicating a small effect, in order to reach a clear treatment advice [45].

For calculating a *z*-score, we used normative data from previous studies. Although there are population norms available for fatigue and depressive symptoms [42, 46], no population norms were available for fear of cancer recurrence as this type of fear is specific for cancer survivors. There were, however, normative data available from a large group of cancer survivors which we could use for this study (M = 10.2, SD = 3.5) [37]. We therefore decided to also use reference groups for our fatigue and depression scores which included cancer survivors. This resulted in the use of fatigue scores from a group of breast cancer survivors (M = 28.47, SD = 13.61) [42]. For the depression scores, we used a reference group which consisted of a group of outpatients who were scheduled for routine office visits with physicians specializing in internal medicine (M = 2.18, SD = 2.96) [39]. In this way, we used normative data that best matched our study population.

#### Treatment advice based on temporal precedence

For the third approach, the symptom which was most useful in predicting the other symptoms determined the treatment advice. This was done by testing Granger causality on the time series of symptoms, assessed with EMA. For this, we used the same protocol as in a previous study, which can be consulted for a detailed description of the analyses [34]. In short, for each individual data set we first checked whether the three symptoms showed sufficient variability to increase the probability of finding a valid vector autoregressive (VAR) model. If a variable did not show sufficient variability (i.e. a mean square successive difference (MSSD) of 50 or less), the variable was excluded from further analyses [47]. Missing measurements were imputed using the Amelia II package in R [48]. Before imputation we checked whether the three symptoms were normally distributed or not. A log or power transformation was performed in the case of right- or left-skewed variables.

After preparing the data, each individual time series was analyzed with “AutoVAR,” an application in R which automatically analyzes the data according to vector autoregressive (VAR) modeling [49–51]. AutoVAR searches for possible VAR models and checks these for validity based on four assumptions (for details, see [49]). If one of the assumptions is not met, the model is adjusted, reestimated and reevaluated until all assumptions are met. We included all eligible symptoms (with a maximum of three) in one VAR model and applied a maximum lag length of 2, indicating a delay of 6 hours. After the analyses, the output was inspected, which consisted of a summary of all the valid VAR models which were found and were presented in a Granger causality image. The symptom which Granger caused the other symptom(s) most consistently (i.e. the symptom which was most useful in forecasting the other symptom(s)) in the highest percentage of valid VAR models was identified. For reasons of clarity, we only considered valid VAR models which showed an overall positive association (e.g. a decrease in fatigue precedes a decrease in depressive symptoms) as with all three treatment advices we aimed to reduce symptoms instead of increasing them. Furthermore, in case two or more associations between symptoms showed an equal percentage (e.g. 50% of the valid models showed a positive association from fatigue to depressive symptoms, but another 50% of the valid models showed a positive association from depressive symptoms to fatigue), we chose the association with the most valid models and with the most consistent (positive) direction of the relationship. In the following cases we labeled the variable 'treatment advice based on temporal precedence' as unclear: no valid models were found, only valid models with negative associations were found, only one variable had a MSSD of > 50, too many assessments were missed which caused problems with imputation, or a patient dropped-out before completing the EMA.

### Statistical analysis

To determine the agreement between the three systematic approaches about treatment advice, we calculated an overall index of agreement according to the Light’s kappa statistic [52, 53]. To achieve this, we first computed a Cohen’s Kappa statistic between each combination of two systematic approaches. Then, we calculated the average mean of these kappa statistics to provide the overall index of agreement. For interpretation, a negative value for kappa, with a maximum of − 1, indicated that agreement was less than the agreement expected by chance (i.e. perfect disagreement). A kappa value of 0 indicated that agreement was not better than chance. Lastly, a kappa value greater than 0 indicated better than chance agreement, with a maximum of + 1 (i.e. perfect agreement). The benchmark scale proposed by Landis and Koch was used to specify the strength of the agreement in the following manner: < 0.00 poor agreement, 0.00–0.20 slight agreement, 0.21–0.40 fair agreement, 0.41–0.60 moderate agreement, 0.61–0.80 substantial agreement and 0.81–1.00 (almost) perfect agreement [54].

### Sensitivity analysis

As previously mentioned, no population norms are available for fear of cancer recurrence for obvious reasons. However, population norms were available for fatigue (M = 22.98, SD = 10.75) and depressive symptoms (M = 1.14, SD = 2.08) [42, 46]. To test for the robustness of our results, we repeated our approach to determine the treatment advice based on symptom severity, using the population norms for fatigue and depressive symptoms in combination with the previous norms for fear of cancer recurrence and recomputed the Light’s kappa statistic accordingly.

## Results

### Participants

The sample selection for this study was finalized on 22 August 2022. Up to that date, 283 cancer survivors were informed about the MATCH study. Of those, 73 patients were excluded and 75 declined. Another 4 patients were still in the screening process. In total, 131 cancer patients were included and randomized in the MATCH study on 22 August 2022. Of those, 69 were randomly allocated to the personalized treatment. Within the personalized treatment arm, 11 participants were eligible to receive one symptom-specific treatment, 24 participants were eligible to receive two treatments and 34 participants were eligible to receive all three symptom-specific treatments. Thus, for this study, we included 58 participants, of which 16 were self-referrals, 39 were referred by their treating physician at the outpatient cancer clinics of an academic hospital or general hospital and 3 were referred by the psycho-oncological mental health care center.

### Descriptive data

The baseline characteristics of the participants are shown in Table 1. Most participants in this study identified as female (71%), were highly educated (57%), had a partner (79%), and previously received psychological or psychiatric care (71%). The average age of the participants was 51.45 years.

**Table 1:** Baseline characteristics of the study participants (*n* = 58)

Sociodemographic characteristics	N (%)
Female	41 (71%)
Mean age in years at entry (SD)	51.45 (11.31)
Education level*	
Low	7 (12%)
Middle	18 (31%)
High	33 (57%)
Having a partner (yes)	46 (79%)
Previously received psychological or psychiatric care (yes)	41 (71%)
Medical characteristics	
Cancer diagnosis	
Breast cancer	24 (41%)
Hematological malignancy	16 (28%)
Esophageal cancer	8 (14%)
Cervical cancer	4 (7%)
Ovarian cancer	2 (3%)
Vulvar cancer	1 (2%)
Bladder and prostate cancer	1 (2%)
Rectal cancer	1 (2%)
Bone cancer	1 (2%)
Type of cancer treatment	
Operation	46 (79%)
Chemotherapy	42 (72%)
Radiotherapy	39 (67%)
Stem cell transplant	11 (19%)
Immunotherapy	9 (16%)
Other treatments	8 (14%)
Mean time since diagnosis in months (SD)	25.76 (17.01)
Mean time since completion of cancer treatment in months (SD)	17.52 (11.74)

*Level of education was categorized as low, middle, or high according to the Dutch National Public Health Compass

With regard to the medical characteristics, most participants had received a breast cancer diagnosis (41%) or a form of a hematological malignancy (28%). Cancer treatment consisted mostly of a combination of an operation, chemotherapy, and/or radiotherapy. On average, patients had received their cancer diagnosis 2 years prior to inclusion and had completed their treatment 1.5 years before inclusion.

### Outcome data

Table 2 presents in detail the treatment advice per systematic approach for patients with specified combinations of co-occurring symptoms.

**Table 2:** Treatment advice per systematic approach according to eligibility

Eligible to receive treatment for (based on the baseline assessment of symptom severity)		Treatment advice per systematic approach		
		*Patient preference*	*Symptom severity*	*Temporal precedence*
Fat + FCR + Dep (*n* = 34)	Fat	17 (50%)	5 (15%)	6 (18%)
	FCR	10 (29%)	16 (47%)	6 (18%)
	Dep	7 (21%)	6 (18%)	11 (32%)
	Unclear	NA	7 (20%)	10 (29%)
	Missing	NA	NA	1 (3%)
Fat + FCR (*n* = 16)	Fat	10 (62%)	6 (38%)	5 (31%)
	FCR	6 (37%)	7 (44%)	0 (0%)
	Dep	0 (0%)	NA	NA
	Unclear	NA	3 (18%)	10 (63%)
	Missing	NA	NA	1 (6%)
Fat + Dep (*n* = 4)	Fat	4 (100%)	2 (50%)	0 (0%)
	FCR	0 (0%)	NA	NA
	Dep	0 (0%)	0 (0%)	0 (0%)
	Unclear	NA	2 (50%)	3 (75%)
	Missing	NA	NA	1 (25%)
FCR + Dep (*n* = 4)	Fat	0 (0%)	NA	NA
	FCR	3 (75%)	2 (50%)	2 (50%)
	Dep	1 (25%)	1 (25%)	0 (0%)
	Unclear	NA	1 (25%)	2 (50%)
	Missing	NA	NA	NA
Overall (*n* = 58)	Fat	31 (53%)	13 (22%)	11 (19%)
	FCR	19 (33%)	25 (43%)	8 (14%)
	Dep	8 (14%)	7 (12%)	11 (19%)
	Unclear	NA	13 (22%)	25 (43%)
	Missing	NA	NA	3 (5%)

Fat, fatigue; FCR, fear of cancer recurrence; Dep, depressive symptoms; NA, not applicable

#### Treatment advice based on patient preference

Overall, 53% of the participants (*n* = 31) preferred treatment for fatigue, 33% (*n* = 19) preferred treatment for fear of cancer recurrence and another 14% (*n* = 8) preferred treatment for depressive symptoms.

#### Treatment advice based on symptom severity

For 22% (*n* = 13), a clear treatment advice could not be formed as the difference between the highest *z*-scores was less than 0.2. For 43% of the participants (*n* = 25), treatment for fear of cancer recurrence was indicated. For 22% of the participants (*n* = 13), treatment for fatigue was indicated and for 12% of the participants (*n* = 7), treatment for depressive symptoms was indicated.

#### Treatment advice based on temporal precedence

**Fig. 1:**
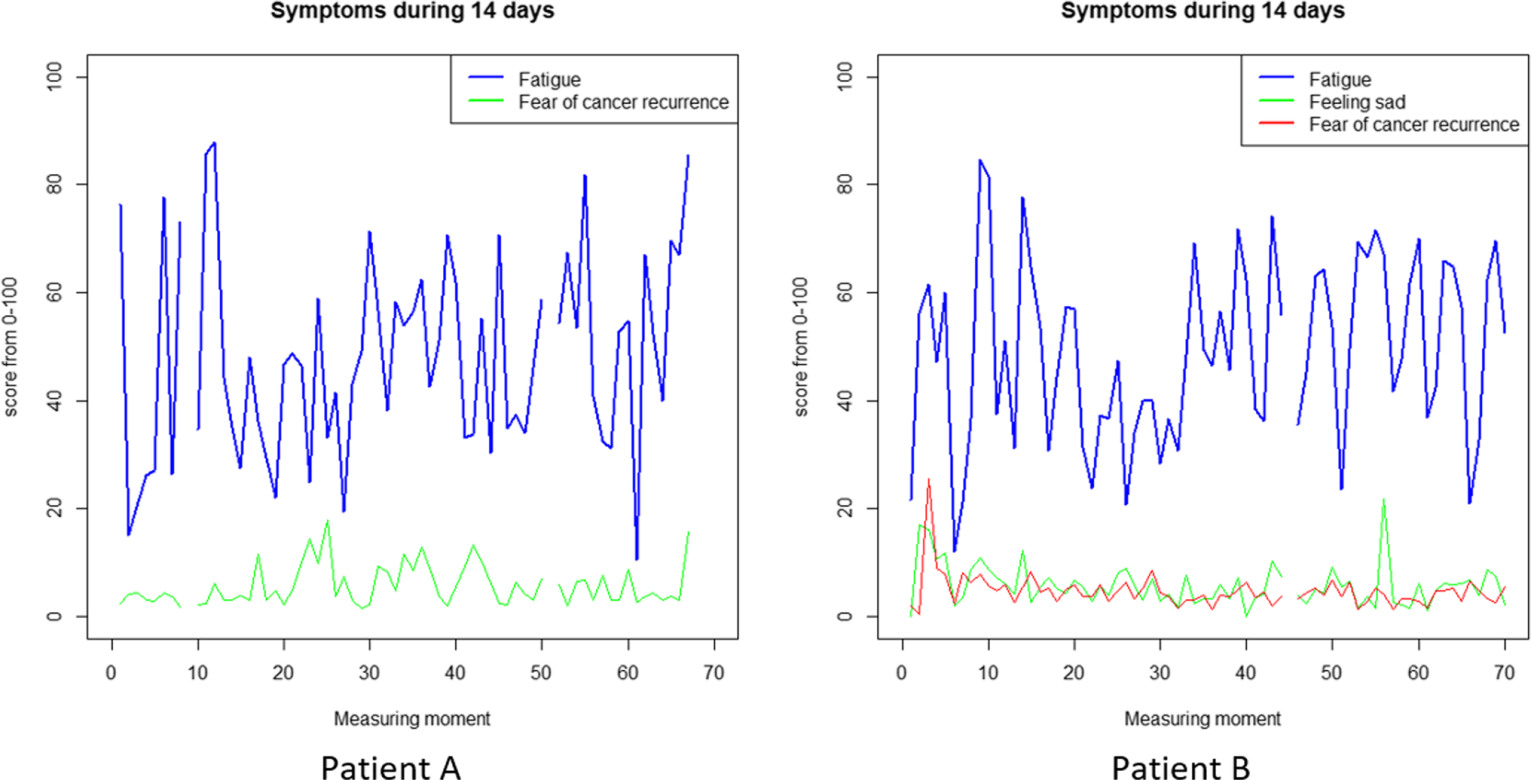
Two examples of temporal variation of two symptoms (patient A) or three symptoms (patient B). In both cases only one symptom showed a MSSD > 50

**Fig. 2:**
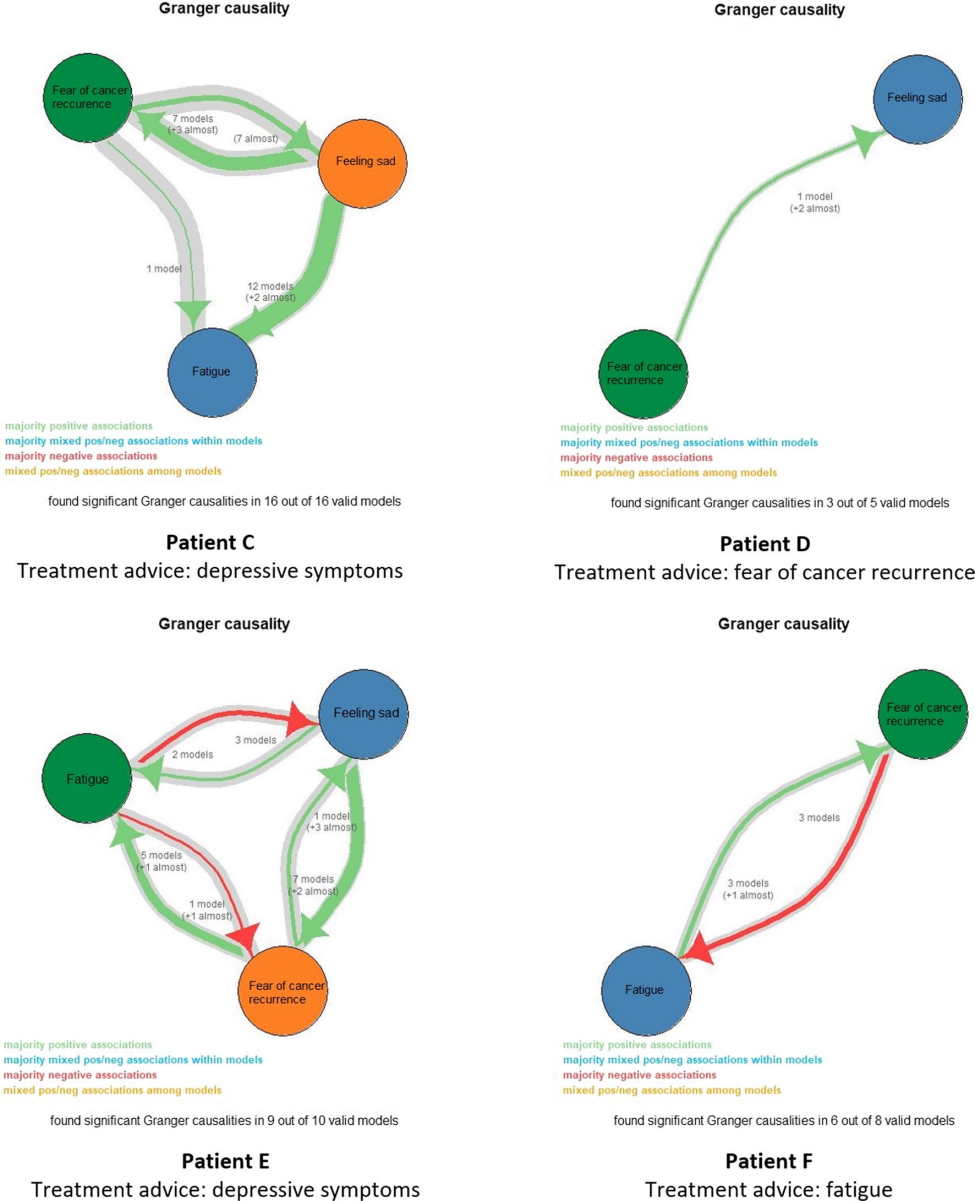
Examples of the Granger causality image in which all valid models and the temporal connections between symptoms are represented. For patient C and D, only positive temporal associations were found (i.e. an increase in one symptom preceded an increase in the other symptom(s)). For patient C, level of depressive symptoms preceded levels of fatigue and fear of cancer recurrence the most often. For patient D, levels of fear of cancer recurrence preceded levels of depressive symptoms. For patients E and F, mixed temporal associations were found (i.e. positive and negative associations)

For 5% of the participants (*n* = 3), temporal precedence could not be established as the data were missing because of pre-treatment drop-out due to cancer recurrence (*n* = 1), symptom reduction (*n* = 1) and a diagnosis of PTSD during intake for which a trauma-specific psychological intervention had priority (*n* = 1). For 43% of the participants (*n* = 25), a clear treatment advice based on temporal precedence could not be formed. This was caused by the following: 1) only one variable showed a MSSD of > 50 (*n* = 9) (see for example patient A and B in Fig. 1); 2) no valid VAR models were found (*n* = 6), 3) valid VAR models were found, but there was no granger causality (*n* = 4); 4) there were too many missing measurements for imputation and in turn to perform the analyses (*n* = 4); and 5) only negative associations were found (*n* = 2).

For 19% of the participants (*n* = 11), treatment for fatigue was indicated and for another 19% (*n* = 11), treatment for depressive symptoms was indicated. For 14% of the participants (*n* = 8), treatment for fear of cancer recurrence was the treatment advice. In Fig. 2 different examples of the Granger causality image are shown, in which all valid models and the temporal connections between symptoms are represented.

### Agreement between approaches

Based on 45 valid cases, there was a moderate agreement between the approach based on patient preference and symptom severity (*k* = 0.53 (95% CI, 0.34 to 0.73), *p* < 0.001). Based on 23 valid cases, there was only a slight agreement between the approach based on symptom severity and temporal precedence (*k* = − 0.04 (95% CI, − 0.21 to 0.14), *p* = 0.72). Based on 30 valid cases, there was also a slight agreement between the approach based on patient preference and temporal precedence (*k* = 0.06 (95% CI, − 0.19 to 0.31), *p* = 0.63). Based on the abovementioned kappa statistics, the overall index of agreement (i.e. Light’s kappa statistic) between the three systematic approaches was 0.19, indicating an overall slight agreement.

When only including the 23 cases in which a clear treatment advice could be formed based on all three systematic approaches, an overall level of agreement of 0.14 was found, still indicating a slight agreement.

### Sensitivity analyses

Based on 38 valid cases, there was a substantial agreement between the treatment advice using cancer survivor specific norms and the population norms (*k* = 0.80 (95% CI, 0.64 to 0.96), *p* < 0.001). The agreement between patient preference and symptom severity improved to substantial agreement based on 46 valid cases (*k* = 0.62 (95% CI, 0.44 to 0.81), *p* < 0.001). There was still a slight agreement between temporal precedence and symptom severity based on 23 valid cases (*k* = 0.0.03 (95% CI, − 0.26 to 0.31), *p* = 0.86). Based on the abovementioned kappa statistics and including the kappa statistic between patient preference and temporal precedence (*k* = 0.06 (95% CI, − 0.19 to 0.31), *p* = 0.63), the overall index of agreement (i.e. Light’s kappa statistic) between the three systematic approaches was 0.24, indicating an overall fair agreement.

When only including the 23 cases in which a clear treatment advice could be formed based on all three systematic approaches an overall level of agreement of 0.25 was found, still indicating fair agreement.

## Discussion

In this study, we investigated the extent to which three different systematic approaches for prioritizing symptoms led to similar treatment advices on which symptom-specific intervention to apply first. We investigated patient preference, symptom severity, and temporal precedence of symptoms in a group of cancer survivors with co-occurring symptoms of cancer-related fatigue, depression and/or fear of cancer recurrence. Based on the kappa statistic, treatment advice based on patient preference and symptom severity showed the most agreement (moderate agreement), especially when we used population norms to determine symptom severity (substantial agreement). This improvement in agreement may be due to the tendency of patients to compare themselves with their healthy selves or with healthy people around them, rather than with other cancer survivors. Treatment advice based on temporal precedence of symptoms deviated the most from the other two approaches, showing only slight agreement. Altogether, the three approaches showed a slight agreement, which increased to fair agreement when using population norms to determine symptom severity.

Our findings show that the three selected approaches lead to different treatment advices, especially with respect to treatment advice based on temporal precedence. This deviation might be explained by patients being less aware of possible temporal relations between symptoms. The most severe symptom could be more apparent for a patient and thereby more likely to be chosen as preferred target in treatment, possibly explaining the highest agreement between these two approaches. In clinical practice, the target of treatment is most often selected in a similar way. However, EMA and investigating temporal precedence can provide additional insights into symptomatology of the individual patient and complement patient’s introspective capability and therapist’s clinical expertise [55, 56].

Although patient preference and symptom severity showed the highest agreement, some differences were observed. Notably, patients most often preferred treatment for fatigue, while based on symptom severity, treatment for fear of cancer recurrence was mostly indicated. Some patients might prefer treatment for a more somatically perceived symptom such as fatigue because of a stigma on mental health problems [57, 58]. In addition, we know that highly fearful patients have a tendency to avoid situations that could raise their anxiety level. In this study, their preference for treatment of fatigue could be part of an avoidant coping style. Although we do not know how participants formed their preference for treatment in this study, we wonder if preference in the abovementioned cases would change after discussing the treatment advices based on the other two approaches, as this could provide new insights for the patient.

For the approach based on symptom severity as well as the approach based on temporal precedence, a clear treatment advice was not always possible. For the approach based on symptom severity, the difference between symptom levels was small in 22% of the cases, indicating equal importance of symptoms. In these cases, patient preference or temporal precedence, could be the deciding factor to determine which symptom to target first. For the approach based on temporal precedence, a clear treatment advice could not be formed in 43% of the cases. Importantly, this approach assumes a temporal order between symptoms, but although symptoms co-occur, they do not necessarily have to be associated. In these cases, patient preference or symptom severity could be the deciding factor to determine which symptom to target first. In addition, sometimes negative associations were found, which in our data was counterintuitive. Discussing the findings of individual symptom networks with the patient is essential to gain a better understanding of the results [55, 56].

Altogether, the three systematic approaches in this study for prioritizing symptoms lead to different treatment advices. Future research should compare the different approaches to determine which approach leads to optimal treatment outcomes and a thoughtful consideration about the aims and feasibility of a chosen approach is recommended. It could be that one approach is better in achieving one treatment outcome or aim over another. For example, if reduction of symptom levels is leading, treatment advice based on symptom severity might be the best option. However, if prevention of drop-out is the most important aim, assessing patient preference might be better. Further, investigating temporal precedence of symptoms could provide new insights into symptomatology of the individual patient. Although not included in this study, other criteria could be applied to select the focus of treatment. For example, one could select the symptom that causes the most severe limitations in daily life. Also, prediction models could be used to calculate which symptom-specific intervention would provide the best treatment outcomes, based on specific characteristics of the individual patient (e.g., [59, 60]). With regard to feasibility, treatment advice based on patient preference was always possible instead of a treatment advice based on symptom severity and temporal precedence. In addition, the experienced burden of the approach on participants is another factor to consider as completing EMA is more time-consuming than the other two approaches. For researchers and clinicians, the analyses for the approach based on temporal precedence and symptom severity are more time-consuming and complex.

## Limitations

In the interpretation of our results, it is important to consider the following limitations. Firstly, patient preference was assessed by asking the patient for which symptom they would like to receive treatment as they experienced this symptom as the most burdensome. This operationalization actually included two questions. We do not know how patients interpreted the question—for some patients, this might have led to the same answer as they wanted help for their most burdensome symptom. However, in case the most burdensome symptom differed from the symptom for which they preferred treatment, it is unknown what led to their answer to this question. For future research, the operationalization of patient preference should be clearer and ideally includes the option to elucidate on how the preference was formed, e.g. through previous experiences with psychological interventions. In the meta-analysis of Swift et al., several operationalizations of patient preference are discussed [25].

A second limitation in this study is the use of our reference groups. Ideally, one should use a reference group consisting of population norms for fear of cancer recurrence, depressive symptoms, and fatigue. However, as fear of cancer recurrence is exclusively present in cancer survivors, this was not possible, and we therefore decided to use norms from reference groups, which included cancer survivors. Preferably, we used the norms of one group of cancer survivors. Unfortunately, to the best of our knowledge, this information was not available. We therefore used the norms from the studies in which the cut-off values of the questionnaires were validated [37–39, 42], and in addition, we checked for the impact of using population norms for fatigue and depression on the overall level of agreement.

A third limitation of this study is the sample size. Our small sample size was appropriate to calculate agreement between different pairs of approaches to prioritize the focus of treatment and to qualitatively interpret the level of agreement between two methods, i.e. “poor agreement” and “fair agreement.” Our sample size was too small, however, to investigate whether the agreement between two different pairs of methods was statistically different from each other. Consequently, we refrained from conducting such an analysis.

Lastly, there are methodological challenges with regard to using EMA and time series analyses based on VAR modeling in clinical practice [61, 62]. In the case of VAR modeling and Granger causality, it only searches for linear relations based on the variables we include in our models. Non-linear associations and external factors are not captured but can influence the results of the analyses. In our study, for example, we also found negative associations between symptoms (i.e. a decrease in one symptom forecasts an increase in the other symptom(s)). The presence of these negative associations was ignored when positive associations were found. Other studies investigating temporal dynamics between symptoms also encountered these findings and speculate on the underlying mechanisms of these negative associations (e.g. [63]). We believe that it could be helpful to discuss the findings with the participants to better understand these negative associations. On a positive note, the most important advantages of EMA are that they are less prone to memory bias and provide more ecologically relevant data [64, 65]. Furthermore, automatizing individual time series analyses can save a lot of time when compared to manual analyses [49], and the consideration of all possible valid models makes results more robust compared to analyses conducted by individual researchers with their own analytical preference [62].

## Conclusion

In conclusion, this study contributes to the development of systematic approaches for selecting the focus of treatment in cancer survivors with co-occurring symptoms of fear of cancer recurrence, depressive symptoms, and fatigue by providing and comparing three different systematic approaches for prioritizing co-occurring symptoms. This study showed that the three approaches for prioritizing symptoms provide different treatment advices. Thus, it matters which approach is chosen, and thoughtful consideration about the aims and feasibility of a chosen approach is recommended. Future research should compare the different approaches to determine which one leads to optimal treatment outcomes, based on the objective(s) it is aiming for and is the most feasible. Until then, we propose to combine the systematic approaches by discussing the results of each approach with the patient in a shared decision-making process to be able to make a well-informed and personalized decision with regard to which symptom to target in psychological treatment.

## Data Availability

After completion of the randomized clinical trial, the datasets generated and/or analyzed during the current study will be available from the corresponding author on reasonable request.
